# Supplementary material for: Life-course neighbourhood deprivation and brain structure in older adults: The Lothian Birth Cohort 1936
Source: medRxiv. 2023 Apr 17:2023.04.13.23288523. Preprint. [Version 1] doi: 10.1101/2023.04.13.23288523 (PMC10153312; doi:10.1101/2023.04.13.23288523)
Supplement: 1 [file NIHPP2023.04.13.23288523v1-supplement-1.pdf]

medRxiv preprint doi: https://doi.org/10.1101/2023.04.13.23288523; this version posted April 17, 2023. The copyright holder for this preprint (which was not certified by peer review) is the author/funder, who has granted medRxiv a license to display the preprint in perpetuity.
It is made available under a CC-BY 4.0 International license .

Supplementary Material

Supplementary Table 1: Pearson's correlation coefficients between neighbourhood deprivation scores and global brain outcomes.
Supplementary Table 2: Relation of main covariates to neighbourhood deprivation scores.
Supplementary Table 3: Number of complete cases for each neighbourhood deprivation exposure and global brain measure pairs.
Supplementary Table 4: Model fit indices for general fractional anisotropy and general mean diffusivity in the main models.
Supplementary Figure 1: Regional associations between life-course models of neighbourhood deprivation and cortical volume in Model 1.
Supplementary Figure 2: Regional associations between life-course models of neighbourhood deprivation and cortical surface area in Model 1.
Supplementary Figure 3: Regional associations between life-course models of neighbourhood deprivation and cortical thickness in Model 1.
Supplementary Figure 4: Regional associations between life-course models of neighbourhood deprivation and cortical volume in Model 2.
Supplementary Figure 5: Regional associations between life-course models of neighbourhood deprivation and cortical surface area in Model 2.
Supplementary Figure 6: Regional associations between life-course models of neighbourhood deprivation and cortical thickness in Model 2.
Supplementary Figure 7: Glass brain plot locating individual white matter tracts used in the analyses.
Supplementary Table 5: Association between life-course models of neighbourhood social deprivation, fractional anisotropy, and mean diffusivity in 12 white matter tracts in Model 1.
Supplementary Table 6: Association between life-course models of neighbourhood social deprivation, fractional anisotropy, and mean diffusivity in 12 white matter tracts in Model 2.
Supplementary Table 7: Interaction of neighbourhood deprivation scores with sex, *APOE* ε4 allele status and adult occupational social class and global brain outcomes.
Supplementary Table 8: Association between life-course models of neighbourhood deprivation and global brain outcomes stratified by adult social class.
Supplementary Table 9: Association between life-course models of neighbourhood deprivation and global brain outcomes after considering exposure during previous epoch.
Supplementary Table 10: Association between life-course models of neighbourhood deprivation and global brain outcomes after adjusting for stroke identified from MRI scans.
Supplementary Table 11: Association between mid-to-late adulthood neighbourhood deprivation and global brain outcomes after adjusting for late adulthood health status.
Supplementary Table 12: Association between life-course models of neighbourhood deprivation and global brain outcomes among those living in Edinburgh throughout each decade of the exposure periods.
Supplementary Table 13: Association between life-course models of neighbourhood deprivation and global brain outcomes among individuals without cognitive impairment.
Supplementary Table 14: Complete case analysis testing the association between life-course models of neighbourhood deprivation and global brain outcomes**.**
Supplementary Figure 8: Pearson's correlation coefficients between individual exposure to neighbourhood deprivation across decades.
Supplementary Figure 9: Directed acyclic graph representing associations between neighbourhood deprivation, global brain outcomes and confounders considered in the main and sensitivity analyses.
Supplementary Table 15: Standardized tract loadings, explained variance and goodness of fir indices for fractional anisotropy and mean diffusivity.

Supplementary Table 1: Pearson's correlation coefficients between neighbourhood deprivation scores and global brain outcomes

	Total brain volume	Grey matter volume	Normal-appearing white matter volume	White matter hyper-intensity volume	Cortical surface area	Mean cortical thickness	General fractional anisotropy	General mean diffusivity	Childhood ND	Young adulthood ND	Mid- to late adulthood ND
Total brain volume											
Grey matter volume	0.92***										
Normal-appearing white matter volume	0.89***	0.75***									
White matter hyperintensity volume	0.06	-0.09*	-0.25***								
Cortical surface area	0.90***	0.80***	0.83***	0.09*							
Mean cortical thickness	0.01	0.21***	-0.10*	-0.14***	-0.16***						
General fractional anisotropy	0.15***	0.20***	0.21***	-0.34***	0.12**	0.27***					
General mean diffusivity	-0.05	-0.09*	-0.19***	0.44***	0.01	-0.12**	-0.53***				
Childhood ND	-0.07	-0.07	-0.04	-0.02	-0.08	-0.06	-0.06	0.00			
Young adulthood ND	-0.03	-0.05	0.01	-0.03	-0.07	-0.15**	-0.14**	-0.04	0.57***		
Mid- to late adulthood ND	-0.15**	-0.17***	-0.15**	0.09	-0.13*	-0.18***	-0.18***	-0.01	0.26***	0.46***	
Accumulated ND	-0.11	-0.12	-0.07	-0.03	-0.12*	-0.16	-0.10	-0.06	0.81***	0.84***	0.69***

Pairwise deletion was applied for missing values to preserve the maximum amount of available information. ND = neighbourhood deprivation.
*** $p < 0.001$
** $p < 0.01$
* $p < 0.05$

Supplementary Table 2: Relation of main covariates to neighbourhood deprivation scores

	Childhood ND		Young adulthood ND		Mid- to late adulthood ND		Accumulated ND	
	Mean (SD)	*p*	Mean (SD)	*p*	Mean (SD)	*p*	Mean (SD)	*p*
Sex								
Male	0.69 (3.51)		-0.84 (2.90)		-2.50 (2.81)		-2.01 (7.19)	
Female	0.44 (3.27)		-0.87 (2.69)		-2.19 (2.65)		-1.99 (6.47)	
Father's occupational social class								
High (professional-managerial)	-0.80 (3.38)	***	-1.79 (2.72)	***	-3.28 (2.40)	***	-5.44 (6.15)	***
Low (skilled, partly skilled and unskilled)	1.06 (3.32)		-0.54 (2.74)		-2.06 (2.76)		-0.93 (6.68)	
APOE ε4 allele status								
ε4 carriers	1.32 (3.25)	*	-0.56 (2.88)		-2.15 (2.90)		0.01 (6.44)	***
Not ε4 carriers	0.23 (3.39)		-1.03 (2.74)		-2.50 (2.61)		-2.96 (6.76)	
Adult occupational social class								
High (professional-managerial)	-0.13 (3.29)	***	-1.32 (2.66)	***	-2.90 (2.56)	***	-3.98 (6.08)	***
Low (skilled, partly skilled and unskilled)	1.48 (3.33)		-0.15 (2.87)		-1.47 (2.79)		0.38 (6.97)	
	Pearson's r	*p*	Pearson's r	*p*	Pearson's r	*p*	Pearson's r	*p*
Childhood IQ	-0.18	**	-0.20	***	-0.23	***	-0.25	***
Years spent in education	-0.27	***	-0.28	***	-0.36	***	-0.42	***

Pairwise deletion was applied for missing values to preserve the maximum amount of available information. Statistical analyses were based on two-sample t-tests for mean differences, and Pearson's correlation for associations between continuous variables. ND = neighbourhood deprivation.

*** $p < 0.001$

** $p < 0.01$

* $p < 0.05$

medRxiv preprint doi: https://doi.org/10.1101/2023.04.13.23288523; this version posted April 17, 2023. The copyright holder for this preprint (which was not certified by peer review) is the author/funder, who has granted medRxiv a license to display the preprint in perpetuity.
It is made available under a CC-BY 4.0 International license .

Supplementary Table 3: Number of complete cases for each neighbourhood deprivation exposure and global brain measure pairs.

	Childhood	Young adulthood	Mid- to late adulthood	Accumulated
Total brain volume (*N*=658)	*n*=305	*n*=376	*n*=390	*n*=276
Grey matter volume (*N*=658)	*n*=305	*n*=376	*n*=390	*n*=276
Normal-appearing white matter volume (*N*=658)	*n*=305	*n*=376	*n*=390	*n*=276
White matter hyperintensity volume (*N*=672)	*n*=311	*n*=383	*n*=396	*n*=281
Cortical surface area (*N*=636)	*n*=296	*n*=367	*n*=379	*n*=268
Mean cortical thickness (*N*=636)	*n*=296	*n*=367	*n*=379	*n*=268
General fractional anisotropy (*N*=665)	*n*=307	*n*=376	*n*=388	*n*=276
General mean diffusivity (*N*=665)	*n*=307	*n*=376	*n*=388	*n*=276

medRxiv preprint doi: https://doi.org/10.1101/2023.04.13.23288523; this version posted April 17, 2023. The copyright holder for this preprint (which was not certified by peer review) is the author/funder, who has granted medRxiv a license to display the preprint in perpetuity.
It is made available under a CC-BY 4.0 International license .

Supplementary Table 4: Model fit indices for general fractional anisotropy and general mean diffusivity in the main models

	Model 1				Model 2			
	CFI	TLI	RMSEA	SRMR	CFI	TLI	RMSEA	SRMR
Childhood neighbourhood deprivation								
General fractional anisotropy	0.95	0.93	0.05	0.04	0.93	0.91	0.06	0.04
General mean diffusivity	0.95	0.94	0.04	0.04	0.93	0.92	0.05	0.04
Young adulthood neighbourhood deprivation								
General fractional anisotropy	0.95	0.93	0.05	0.04	0.94	0.93	0.04	0.04
General mean diffusivity	0.93	0.91	0.06	0.04	0.93	0.91	0.05	0.04
Mid- to late adulthood neighbourhood deprivation								
General fractional anisotropy	0.94	0.92	0.05	0.04	0.94	0.93	0.04	0.04
General mean diffusivity	0.93	0.91	0.06	0.05	0.92	0.91	0.05	0.04
Accumulated neighbourhood deprivation								
General fractional anisotropy	0.94	0.93	0.05	0.04	0.94	0.93	0.04	0.04
General mean diffusivity	0.93	0.91	0.06	0.04	0.93	0.91	0.05	0.04

Reference values are: CFI>0.95, TLI>0.95, RMSEA<0.06, SRMR<0.08 (1). CFI=Comparative Fit Index; TLI= Tucker-Lewis Index; RMSEA=Root Mean Square Error of Approximation; SRMR=Standardized Root Mean Square Residual

medRxiv preprint doi: https://doi.org/10.1101/2023.04.13.23288523; this version posted April 17, 2023. The copyright holder for this preprint (which was not certified by peer review) is the author/funder, who has granted medRxiv a license to display the preprint in perpetuity.
It is made available under a CC-BY 4.0 International license .

Supplementary Figure 1: Regional associations between life-course models of neighbourhood deprivation and cortical volume in Model 1. Standardized coefficients were obtained in linear regression models fitted within the structural equation modelling framework applying full information maximum likelihood estimation. Sample size was *N*=622; pairwise complete observations were *n*=289, *n*=358, *n*=371 and *n*=262 for childhood, young adulthood, mid-to-late adulthood, and accumulated neighbourhood deprivation (ND), respectively. Models were adjusted for sex, age, and intracranial volume. Heatmaps show (from left to right): standardised betas and false discovery rate adjusted *p*-values (p_{FDR} < 0.05). The non-cortical mask is shown in black.

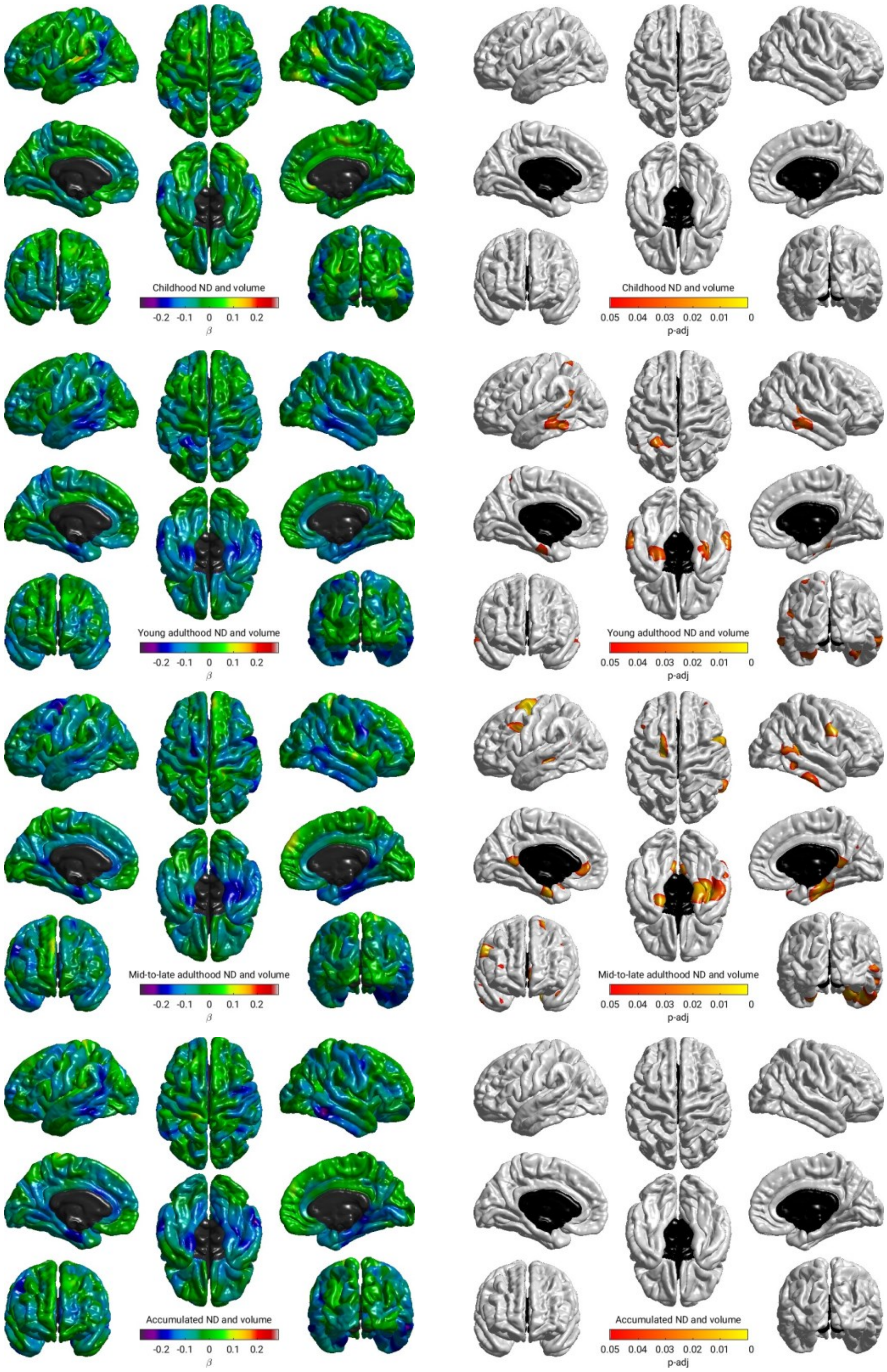

medRxiv preprint doi: https://doi.org/10.1101/2023.04.13.23288523; this version posted April 17, 2023. The copyright holder for this preprint (which was not certified by peer review) is the author/funder, who has granted medRxiv a license to display the preprint in perpetuity.
It is made available under a CC-BY 4.0 International license .

Supplementary Figure 2: Regional associations between life-course models of neighbourhood deprivation and cortical surface area in Model 1. Standardized coefficients were obtained in linear regression models fitted within the structural equation modelling framework applying full information maximum likelihood estimation. Sample size was *N*=622; pairwise complete observations were *n*=289, *n*=358, *n*=371 and *n*=262 for childhood, young adulthood, mid-to-late adulthood, and accumulated neighbourhood deprivation (ND), respectively. Models were adjusted for sex, age, and intracranial volume. Heatmaps show (from left to right): standardised betas and false discovery rate adjusted *p*-values ($p_{FDR} < 0.05$). The non-cortical mask is shown in black.

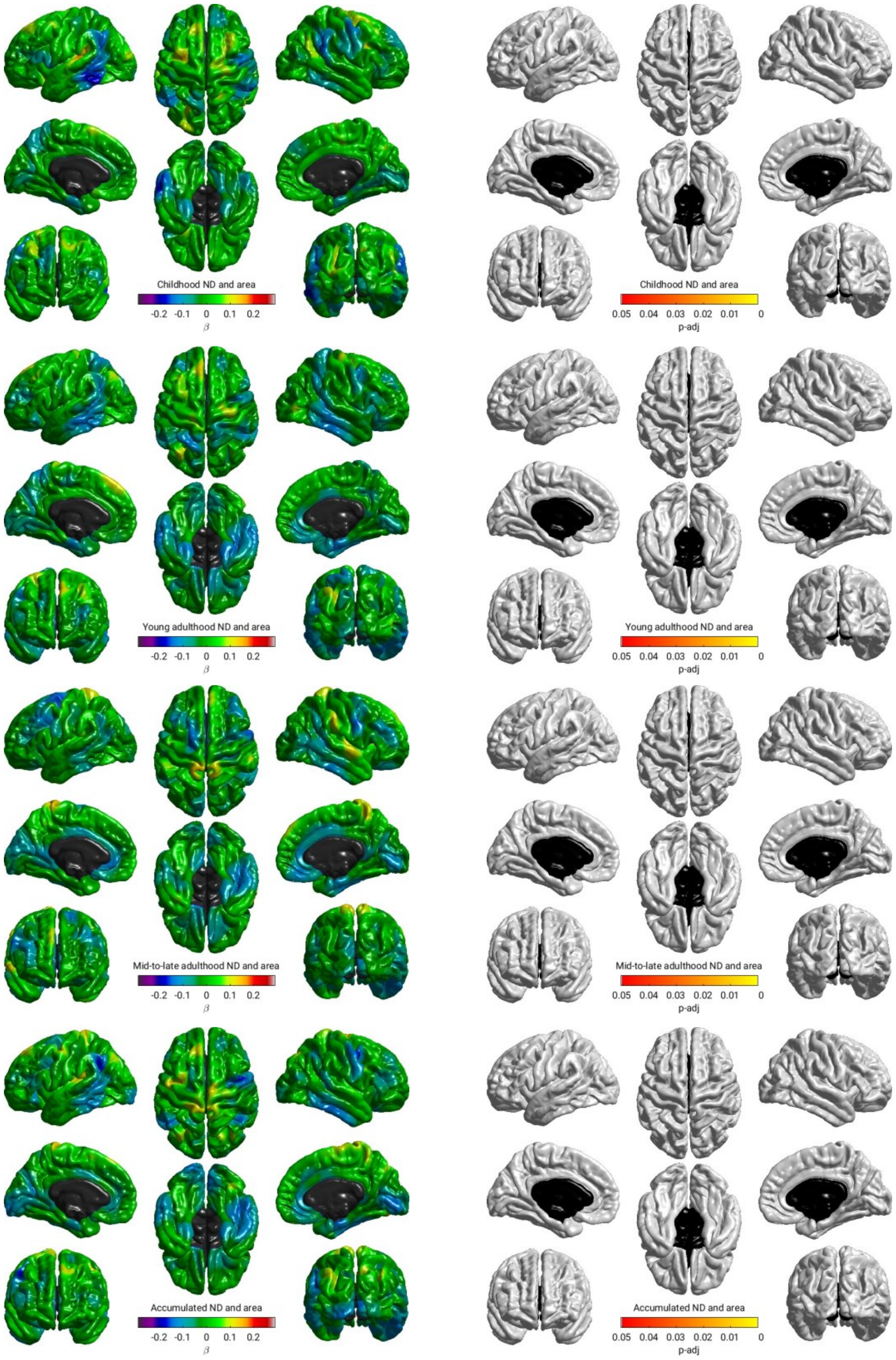

medRxiv preprint doi: https://doi.org/10.1101/2023.04.13.23288523; this version posted April 17, 2023. The copyright holder for this preprint (which was not certified by peer review) is the author/funder, who has granted medRxiv a license to display the preprint in perpetuity.
It is made available under a CC-BY 4.0 International license .

Supplementary Figure 3: Regional associations between life-course models of neighbourhood deprivation and cortical thickness in Model 1. Standardized coefficients were obtained in linear regression models fitted within the structural equation modelling framework applying full information maximum likelihood estimation. Sample size was N=622; pairwise complete observations were n=289, n=358, n=371 and n=262 for childhood, young adulthood, mid-to-late adulthood, and accumulated neighbourhood deprivation (ND), respectively. Models were adjusted for sex, age, and intracranial volume. Heatmaps show (from left to right): standardised betas and false discovery rate adjusted p-values ($p_{FDR} < 0.05$). The non-cortical mask is shown in black.

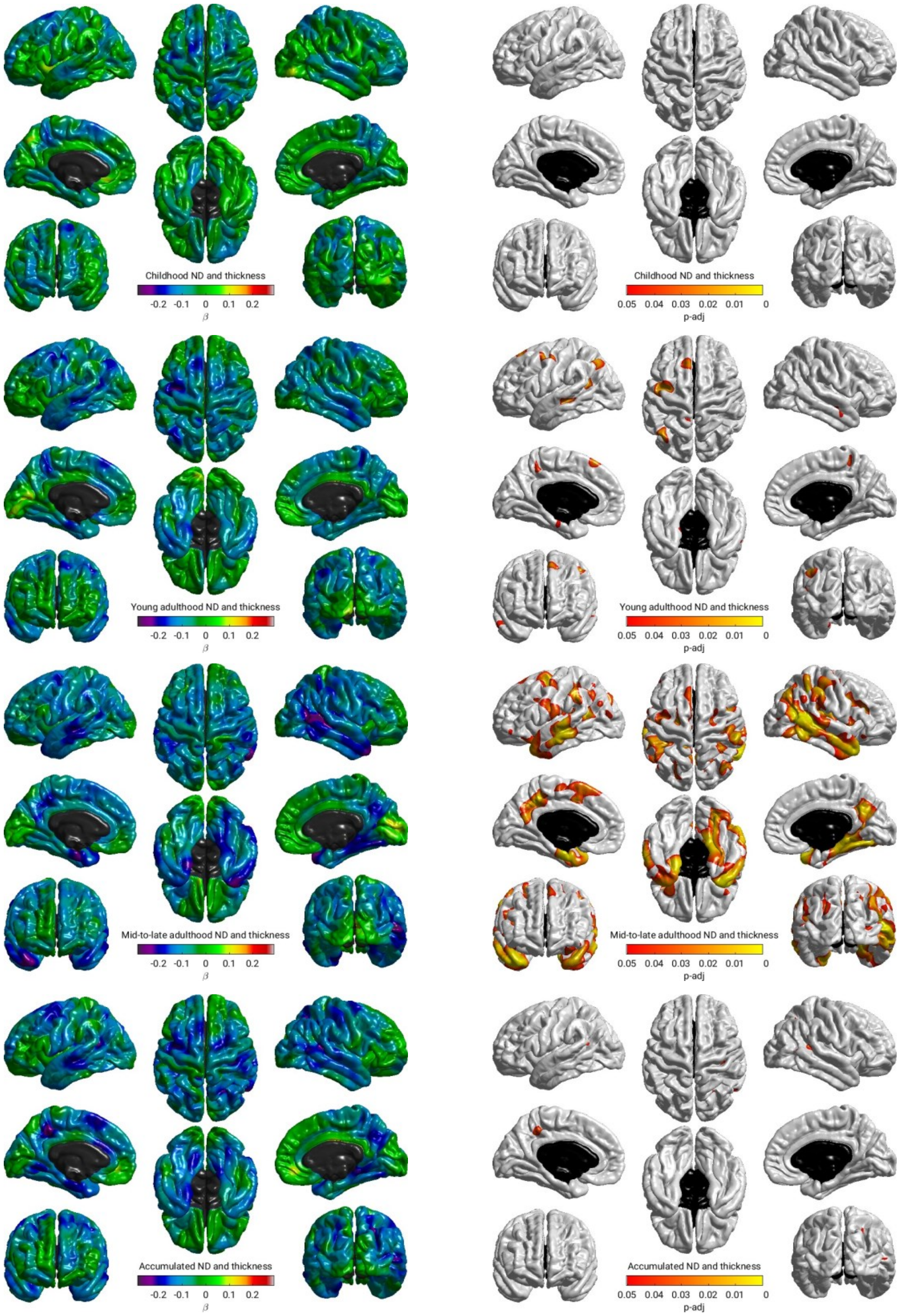

medRxiv preprint doi: https://doi.org/10.1101/2023.04.13.23288523; this version posted April 17, 2023. The copyright holder for this preprint (which was not certified by peer review) is the author/funder, who has granted medRxiv a license to display the preprint in perpetuity.
It is made available under a CC-BY 4.0 International license .

Supplementary Figure 4: Regional associations between life-course models of neighbourhood deprivation and cortical volume in Model 2. Standardized coefficients were obtained in linear regression models fitted within the structural equation modelling framework applying full information maximum likelihood estimation. Sample size was N=622; pairwise complete observations were n=289, n=358, n=371 and n=262 for childhood, young adulthood, mid-to-late adulthood, and accumulated neighbourhood deprivation (ND), respectively. Models were adjusted for sex, age, intracranial volume, father's occupational social class, APOE ε4 allele status. In addition, young adulthood models were adjusted for childhood IQ and years spent in education, and mid- to late adulthood/ accumulation models also for adult occupational social class. Heatmaps show (from left to right): standardised betas and false discovery rate adjusted p-values ($p_{FDR} < 0.05$). The non-cortical mask is shown in black.

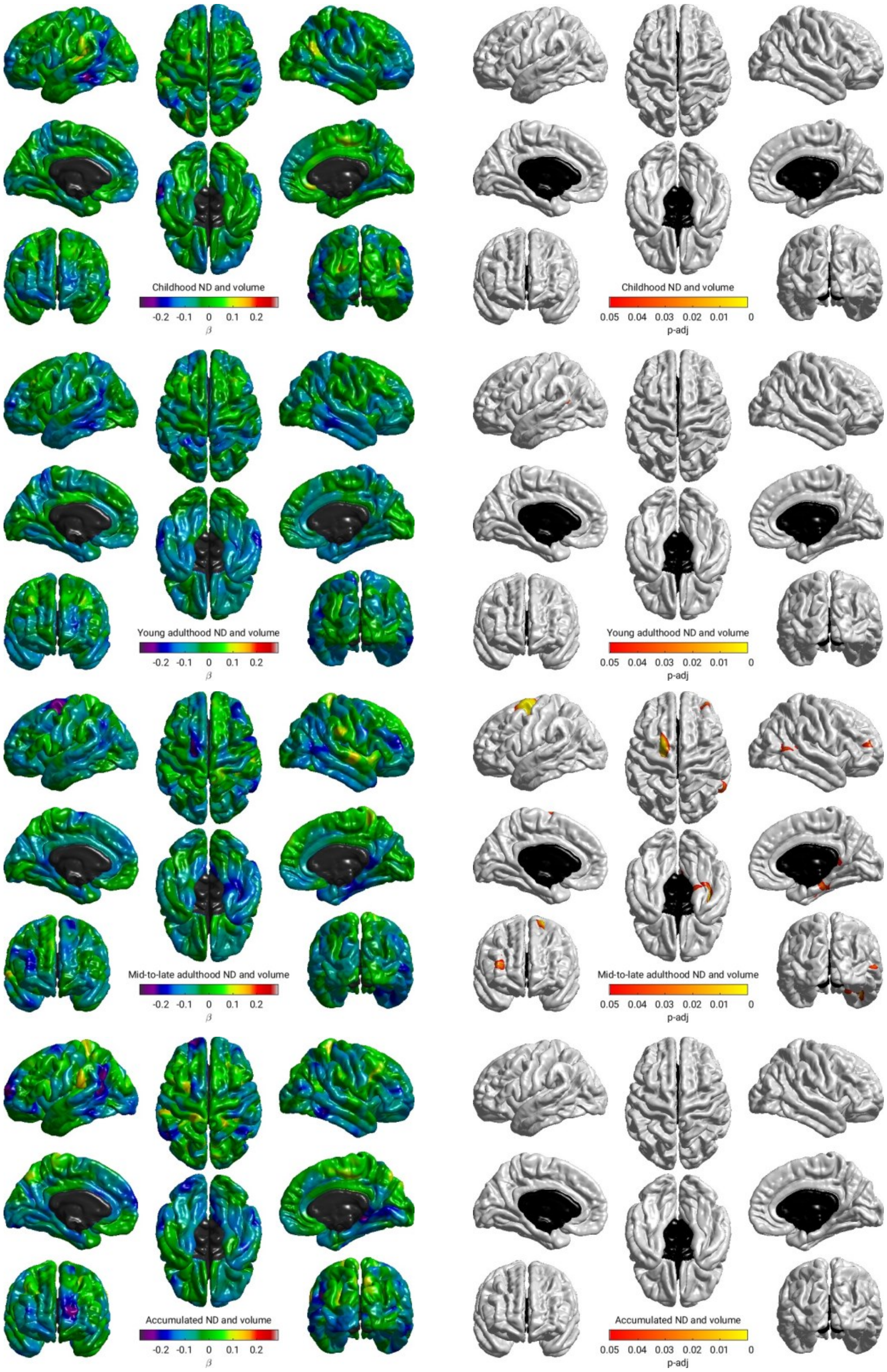

medRxiv preprint doi: https://doi.org/10.1101/2023.04.13.23288523; this version posted April 17, 2023. The copyright holder for this preprint (which was not certified by peer review) is the author/funder, who has granted medRxiv a license to display the preprint in perpetuity.
It is made available under a CC-BY 4.0 International license .

Supplementary Figure 5: Regional associations between life-course models of neighbourhood deprivation and cortical surface area in Model 2. Standardized coefficients were obtained in linear regression models fitted within the structural equation modelling framework applying full information maximum likelihood estimation. Sample size was *N*=622; pairwise complete observations were *n*=289, *n*=358, *n*=371 and *n*=262 for childhood, young adulthood, mid-to-late adulthood, and accumulated neighbourhood deprivation (ND), respectively. Models were adjusted for sex, age, intracranial volume, father's occupational social class, APOE ε4 allele status. In addition, young adulthood models were adjusted for childhood IQ and years spent in education, and mid- to late adulthood/ accumulation models also for adult occupational social class. Heatmaps show (from left to right): standardised betas and false discovery rate adjusted *p*-values ($p_{FDR} < 0.05$). The non-cortical mask is shown in black.

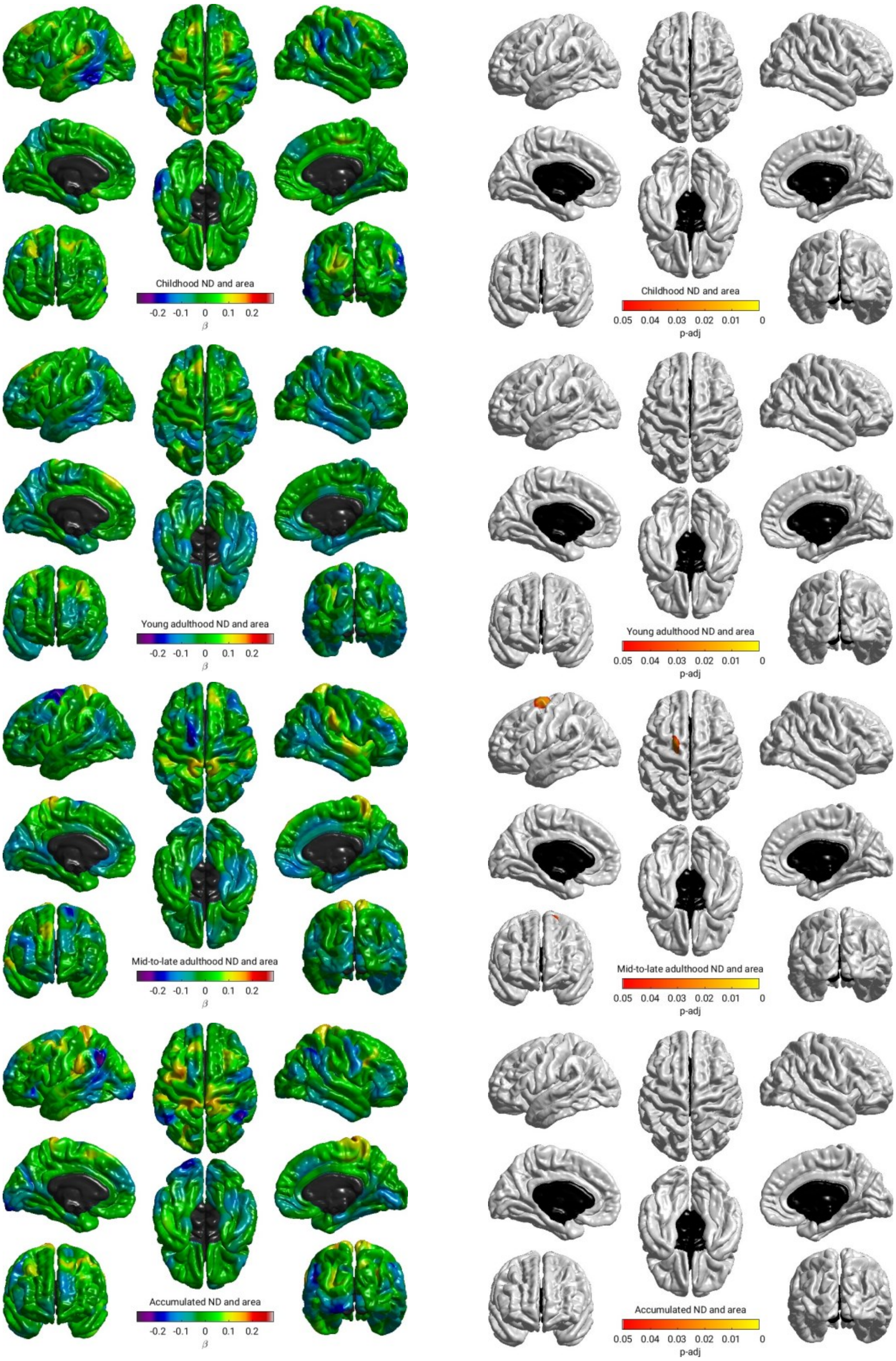

medRxiv preprint doi: https://doi.org/10.1101/2023.04.13.23288523; this version posted April 17, 2023. The copyright holder for this preprint (which was not certified by peer review) is the author/funder, who has granted medRxiv a license to display the preprint in perpetuity.
It is made available under a CC-BY 4.0 International license .

Supplementary Figure 6: Regional associations between life-course models of neighbourhood deprivation and cortical thickness in Model 2. Standardized coefficients were obtained in linear regression models fitted within the structural equation modelling framework applying full information maximum likelihood estimation. Sample size was N=622; pairwise complete observations were n=289, n=358, n=371 and n=262 for childhood, young adulthood, mid-to-late adulthood, and accumulated neighbourhood deprivation (ND), respectively. Models were adjusted for sex, age, intracranial volume, father's occupational social class, APOE ε4 allele status. In addition, young adulthood models were adjusted for childhood IQ and years spent in education, and mid- to late adulthood/ accumulation models also for adult occupational social class. Heatmaps show (from left to right): standardised betas and false discovery rate adjusted p-values (p_{FDR} < 0.05). The non-cortical mask is shown in black.

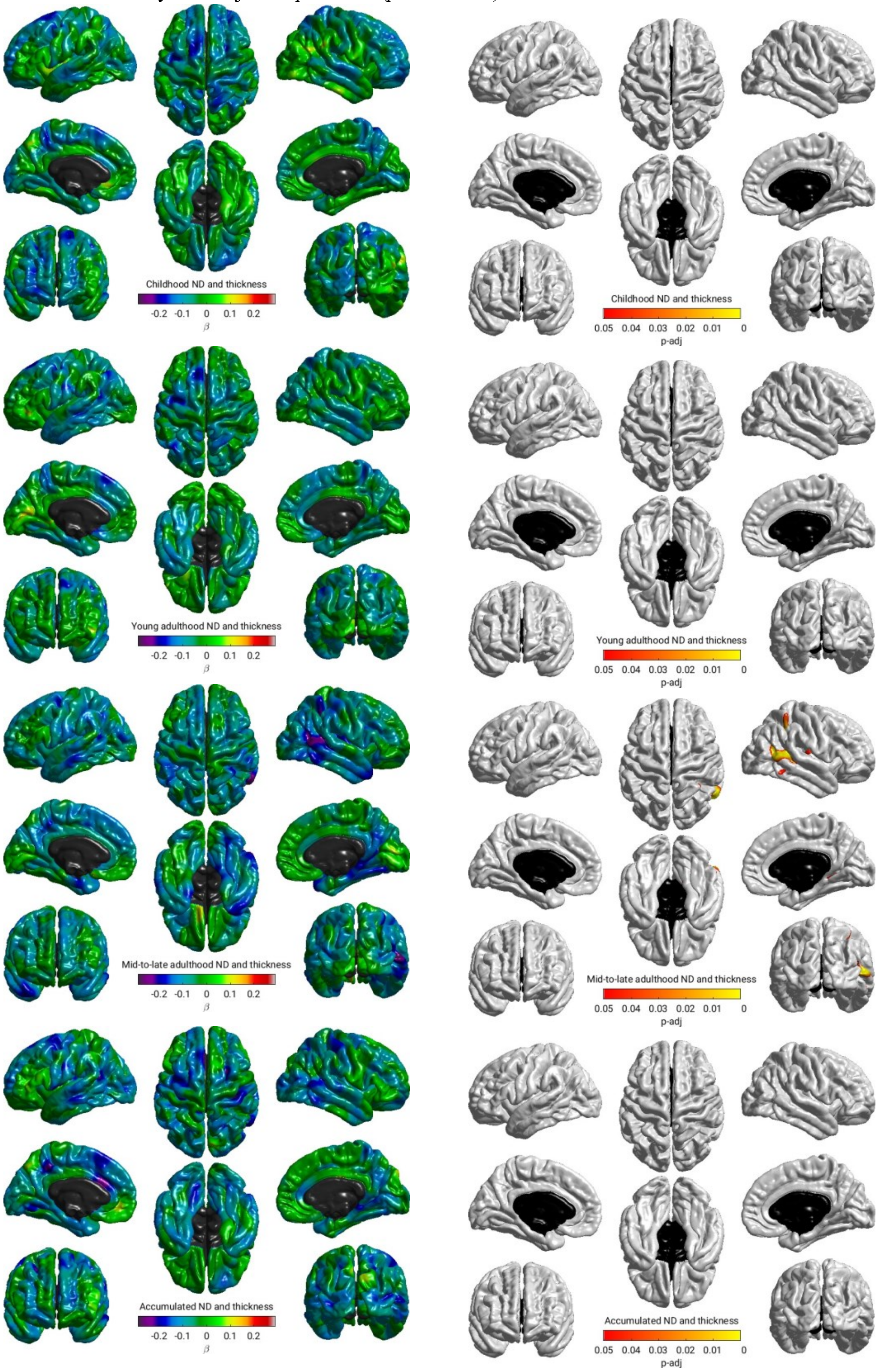

Supplementary Figure 7: Glass brain plot locating white matter tracts used in the analyses. The ventral cingulum, coloured in grey, was not included in analyses as the rostral and ventral cingula are subdivisions of the same tract (an approach adopted previously in this cohort, e.g., (2))

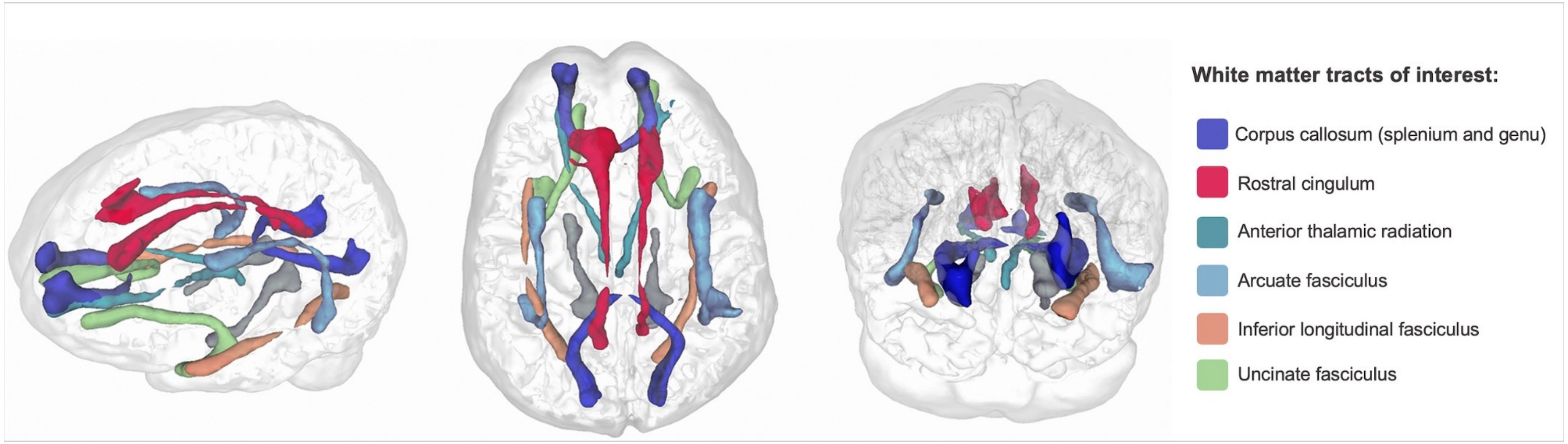

medRxiv preprint doi: https://doi.org/10.1101/2023.04.13.23288523; this version posted April 17, 2023. The copyright holder for this preprint (which was not certified by peer review) is the author/funder, who has granted medRxiv a license to display the preprint in perpetuity. It is made available under a CC-BY 4.0 International license .

Supplementary Table 5: Association between life-course models of neighbourhood deprivation, fractional anisotropy and mean diffusivity in 12 white matter tracts in Model 1

	Fractional anisotropy				Mean diffusivity			
	β	SE	*p*	p_{FDR}	*β*	SE	*p*	p_{FDR}
Childhood neighbourhood deprivation (n=279 to 306)								
Genu of corpus callosum	0.04	0.06	0.54	0.82	-0.02	0.06	0.72	0.95
Splenium of corpus callosum	-0.06	0.06	0.28	0.82	0.06	0.06	0.31	0.95
Left arcuate fasciculus	-0.03	0.05	0.61	0.82	0.00	0.06	0.99	0.99
Right arcuate fasciculus	-0.04	0.06	0.47	0.82	0.03	0.06	0.59	0.95
Left anterior thalamic radiation	-0.07	0.06	0.22	0.82	0.00	0.06	0.98	0.99
Right anterior thalamic radiation	0.00	0.06	0.97	0.97	-0.09	0.06	0.13	0.90
Left rostral cingulum	-0.09	0.06	0.13	0.82	0.03	0.06	0.64	0.95
Right rostral cingulum	-0.01	0.05	0.86	0.94	-0.03	0.06	0.61	0.95
Left inferior longitudinal fasciculus	0.02	0.06	0.68	0.82	-0.04	0.07	0.56	0.95
Right inferior longitudinal fasciculus	0.03	0.05	0.56	0.82	-0.08	0.06	0.15	0.90
Left uncinate fasciculus	-0.07	0.06	0.27	0.82	0.03	0.06	0.59	0.95
Right uncinate fasciculus	-0.03	0.06	0.63	0.82	0.02	0.06	0.79	0.95
Young adulthood neighbourhood deprivation (n=346 to 375)								
Genu of corpus callosum	-0.01	0.05	0.80	0.87	-0.08	0.05	0.15	0.58
Splenium of corpus callosum	0.00	0.05	0.95	0.95	-0.02	0.05	0.70	0.79
Left arcuate fasciculus	-0.09	0.05	0.06	0.25	-0.06	0.05	0.26	0.64
Right arcuate fasciculus	-0.03	0.05	0.51	0.62	-0.01	0.06	0.80	0.80
Left anterior thalamic radiation	-0.03	0.05	0.52	0.62	-0.10	0.05	0.05	0.58
Right anterior thalamic radiation	-0.05	0.05	0.30	0.51	-0.08	0.05	0.15	0.58
Left rostral cingulum	-0.07	0.05	0.21	0.51	-0.03	0.05	0.57	0.79
Right rostral cingulum	-0.04	0.05	0.40	0.59	-0.06	0.05	0.27	0.64
Left inferior longitudinal fasciculus	-0.06	0.05	0.30	0.51	0.06	0.06	0.37	0.73
Right inferior longitudinal fasciculus	-0.09	0.05	0.06	0.25	0.02	0.06	0.69	0.79
Left uncinate fasciculus	-0.11	0.05	0.04	0.25	-0.02	0.05	0.72	0.79
Right uncinate fasciculus	-0.09	0.05	0.08	0.25	0.02	0.06	0.73	0.79
Mid- to late adulthood neighbourhood deprivation (n=359 to 387)								
Genu of corpus callosum	0.02	0.05	0.68	0.74	-0.08	0.05	0.13	0.39
Splenium of corpus callosum	**-0.14**	**0.05**	**0.01**	**0.03**	0.08	0.06	0.16	0.39
Left arcuate fasciculus	**-0.13**	**0.05**	**0.01**	**0.03**	0.03	0.05	0.55	0.70
Right arcuate fasciculus	-0.11	0.05	0.05	0.12	0.00	0.06	0.97	0.97
Left anterior thalamic radiation	-0.08	0.05	0.12	0.17	-0.08	0.05	0.12	0.39
Right anterior thalamic radiation	**-0.17**	**0.05**	**0.001**	**0.01**	0.09	0.05	0.11	0.39
Left rostral cingulum	-0.08	0.05	0.11	0.17	-0.01	0.05	0.90	0.97
Right rostral cingulum	-0.01	0.05	0.88	0.88	-0.03	0.05	0.57	0.70
Left inferior longitudinal fasciculus	-0.08	0.05	0.11	0.17	0.05	0.06	0.38	0.70
Right inferior longitudinal fasciculus	**-0.13**	**0.05**	**0.01**	**0.03**	0.03	0.06	0.59	0.70
Left uncinate fasciculus	-0.08	0.05	0.13	0.17	-0.08	0.05	0.16	0.39
Right uncinate fasciculus	-0.05	0.05	0.38	0.46	-0.04	0.06	0.45	0.70
Accumulated neighbourhood deprivation (n=251 to 275)								
Genu of corpus callosum	0.04	0.06	0.54	0.66	-0.08	0.06	0.22	0.69
Splenium of corpus callosum	-0.09	0.06	0.11	0.37	0.05	0.06	0.41	0.69
Left arcuate fasciculus	-0.04	0.06	0.51	0.66	-0.07	0.06	0.25	0.69
Right arcuate fasciculus	-0.04	0.06	0.55	0.66	-0.02	0.07	0.78	0.85
Left anterior thalamic radiation	-0.04	0.06	0.51	0.66	-0.09	0.06	0.14	0.69
Right anterior thalamic radiation	-0.09	0.06	0.12	0.37	-0.05	0.06	0.39	0.69
Left rostral cingulum	-0.12	0.06	0.06	0.37	0.01	0.06	0.93	0.93
Right rostral cingulum	0.02	0.06	0.7	0.77	-0.08	0.06	0.18	0.69
Left inferior longitudinal fasciculus	-0.07	0.06	0.28	0.6	0.05	0.07	0.51	0.69
Right inferior longitudinal fasciculus	-0.09	0.06	0.1	0.37	-0.04	0.06	0.46	0.69
Left uncinate fasciculus	-0.07	0.06	0.3	0.6	-0.03	0.07	0.65	0.78
Right uncinate fasciculus	-0.01	0.06	0.84	0.84	-0.05	0.07	0.46	0.69

Models were fitted within the structural equation modelling framework applying full information maximum likelihood estimation. Total sample size was *N*=633 for genu of corpus callosum, *N*=652 for splenium of corpus callosum, *N*=655 left arcuate fasciculus, and *N*=621 for right arcuate fasciculus, *N*=643 for left anterior thalamic radiation, *N*=662 right

medRxiv preprint doi: https://doi.org/10.1101/2023.04.13.23288523; this version posted April 17, 2023. The copyright holder for this preprint (which was not certified by peer review) is the author/funder, who has granted medRxiv a license to display the preprint in perpetuity.
It is made available under a CC-BY 4.0 International license .

anterior thalamic radiation, *N*=647 for left rostral cingulum, *N*=643 for right rostral cingulum, *N*=663 for left inferior longitudinal fasciculus, *N*=663 for left and right inferior longitudinal fasciculus, *N*=618 for left uncinate fasciculus and *N*=647 for right uncinate fasciculus; this table indicates ranges of observations for each exposure-outcomes pairs on which reported effect sizes are based (*n*). Bold typeface denotes false discovery rate adjusted significance (p_{FDR}). SE = standard error.

All models were adjusted for sex and age.

medRxiv preprint doi: https://doi.org/10.1101/2023.04.13.23288523; this version posted April 17, 2023. The copyright holder for this preprint (which was not certified by peer review) is the author/funder, who has granted medRxiv a license to display the preprint in perpetuity.
It is made available under a CC-BY 4.0 International license .

Supplementary Table 6: Association between life-course models of neighbourhood deprivation, fractional anisotropy and mean diffusivity in 12 white matter tracts in Model 2

	Fractional anisotropy				Mean diffusivity			
	β	SE	p	p_{FDR}	β	SE	p	p_{FDR}
Childhood neighbourhood deprivation (n=279 to 306)								
Genu of corpus callosum	0.01	0.06	0.89	0.89	0.01	0.06	0.86	0.97
Splenium of corpus callosum	-0.07	0.06	0.21	0.66	0.09	0.06	0.12	0.84
Left arcuate fasciculus	-0.02	0.06	0.69	0.89	-0.01	0.06	0.81	0.97
Right arcuate fasciculus	-0.04	0.06	0.47	0.89	0.03	0.07	0.61	0.97
Left anterior thalamic radiation	-0.08	0.06	0.18	0.66	0.00	0.06	0.99	0.99
Right anterior thalamic radiation	0.01	0.06	0.86	0.89	-0.09	0.06	0.14	0.84
Left rostral cingulum	-0.10	0.06	0.10	0.66	0.03	0.06	0.64	0.97
Right rostral cingulum	-0.03	0.06	0.66	0.89	-0.01	0.06	0.89	0.97
Left inferior longitudinal fasciculus	0.03	0.06	0.61	0.89	-0.04	0.07	0.61	0.97
Right inferior longitudinal fasciculus	0.01	0.06	0.86	0.89	-0.07	0.06	0.23	0.90
Left uncinate fasciculus	-0.08	0.06	0.22	0.66	0.04	0.06	0.48	0.97
Right uncinate fasciculus	-0.03	0.06	0.63	0.89	0.03	0.06	0.67	0.97
Young adulthood neighbourhood deprivation (n=346 to 375)								
Genu of corpus callosum	-0.05	0.06	0.34	0.51	-0.05	0.06	0.39	0.73
Splenium of corpus callosum	-0.01	0.05	0.82	0.82	0.00	0.06	0.93	0.93
Left arcuate fasciculus	-0.10	0.05	0.07	0.27	-0.06	0.05	0.25	0.60
Right arcuate fasciculus	-0.04	0.06	0.43	0.57	-0.02	0.06	0.77	0.92
Left anterior thalamic radiation	-0.03	0.06	0.62	0.67	-0.12	0.06	0.03	0.39
Right anterior thalamic radiation	-0.04	0.06	0.48	0.58	-0.09	0.06	0.12	0.50
Left rostral cingulum	-0.06	0.06	0.29	0.50	-0.04	0.06	0.49	0.73
Right rostral cingulum	-0.07	0.05	0.17	0.35	-0.07	0.05	0.22	0.60
Left inferior longitudinal fasciculus	-0.08	0.06	0.15	0.35	0.11	0.07	0.11	0.50
Right inferior longitudinal fasciculus	-0.12	0.05	0.02	0.13	0.05	0.06	0.43	0.73
Left uncinate fasciculus	-0.14	0.06	0.01	0.13	-0.01	0.06	0.85	0.92
Right uncinate fasciculus	-0.09	0.06	0.09	0.27	0.02	0.06	0.78	0.92
Mid- to late adulthood neighbourhood deprivation (n=359 to 387)								
Genu of corpus callosum	-0.03	0.06	0.65	0.65	-0.03	0.06	0.57	0.74
Splenium of corpus callosum	**-0.19**	**0.06**	**0.001**	**0.003**	0.15	0.06	0.01	0.18
Left arcuate fasciculus	**-0.16**	**0.05**	**0.004**	**0.01**	0.08	0.06	0.14	0.41
Right arcuate fasciculus	**-0.16**	**0.06**	**0.009**	**0.02**	0.05	0.06	0.42	0.62
Left anterior thalamic radiation	-0.09	0.06	0.12	0.18	-0.07	0.06	0.22	0.44
Right anterior thalamic radiation	**-0.19**	**0.06**	**0.001**	**0.003**	0.12	0.06	0.04	0.24
Left rostral cingulum	-0.07	0.06	0.21	0.28	-0.01	0.06	0.84	0.84
Right rostral cingulum	-0.05	0.06	0.42	0.46	-0.02	0.06	0.75	0.82
Left inferior longitudinal fasciculus	-0.12	0.06	0.04	0.08	0.12	0.07	0.06	0.24
Right inferior longitudinal fasciculus	**-0.18**	**0.06**	**0.001**	**0.003**	0.08	0.06	0.19	0.44
Left uncinate fasciculus	-0.11	0.06	0.05	0.09	-0.05	0.06	0.38	0.62
Right uncinate fasciculus	-0.05	0.06	0.41	0.46	-0.03	0.06	0.62	0.74
Accumulated neighbourhood deprivation (n=251 to 275)								
Genu of corpus callosum	-0.02	0.07	0.82	0.95	-0.05	0.07	0.50	0.73
Splenium of corpus callosum	-0.18	0.07	0.01	0.10	0.17	0.07	0.02	0.20
Left arcuate fasciculus	-0.05	0.07	0.44	0.66	-0.04	0.07	0.55	0.73
Right arcuate fasciculus	-0.08	0.07	0.28	0.47	0.03	0.08	0.69	0.83
Left anterior thalamic radiation	-0.04	0.07	0.53	0.71	-0.14	0.07	0.05	0.30
Right anterior thalamic radiation	-0.11	0.07	0.10	0.28	-0.04	0.07	0.55	0.73
Left rostral cingulum	-0.12	0.07	0.09	0.28	-0.02	0.07	0.82	0.84
Right rostral cingulum	-0.01	0.07	0.87	0.95	-0.08	0.07	0.22	0.66
Left inferior longitudinal fasciculus	-0.10	0.07	0.16	0.32	0.14	0.08	0.10	0.42
Right inferior longitudinal fasciculus	-0.15	0.07	0.02	0.13	0.01	0.07	0.84	0.84
Left uncinate fasciculus	-0.12	0.08	0.12	0.28	-0.05	0.08	0.55	0.73
Right uncinate fasciculus	0.00	0.07	0.96	0.96	-0.07	0.08	0.35	0.73

Models were fitted within the structural equation modelling framework applying full information maximum likelihood estimation. Total sample size was N=633 for genu of corpus callosum, N=652 for splenium of corpus callosum, N=655 left arcuate fasciculus, and N=621 for right arcuate fasciculus, N=643 for left anterior thalamic radiation, N=662 right

medRxiv preprint doi: https://doi.org/10.1101/2023.04.13.23288523; this version posted April 17, 2023. The copyright holder for this preprint (which was not certified by peer review) is the author/funder, who has granted medRxiv a license to display the preprint in perpetuity.
It is made available under a CC-BY 4.0 International license .

anterior thalamic radiation, *N*=647 for left rostral cingulum, *N*=643 for right rostral cingulum, *N*=663 for left inferior longitudinal fasciculus, *N*=663 for left and right inferior longitudinal fasciculus, *N*=618 for left uncinate fasciculus and *N*=647 for right uncinate fasciculus; this table indicates ranges of observations for each exposure-outcomes pairs on which reported effect sizes are based (*n*). Bold typeface denotes false discovery rate adjusted significance (p_{FDR}). SE = standard error.

Models were adjusted for sex, age, father's occupational social class and *APOE* ε4 allele status. In addition, young adulthood models were adjusted for childhood IQ and years spent in education and mid- to late adulthood/ accumulation models also for adult occupational social class.

Supplementary Table 7: Interaction of neighbourhood deprivation with sex, *APOE* ε4 allele status and adult occupational social class and global brain outcomes

	Sex				*APOE* ε4 allele status				Father's social class				Adult social class			
	β	SE	p	p_{FDR}	β	SE	p	p_{FDR}	β	SE	p	p_{FDR}	β	SE	p	p_{FDR}
Childhood neighbourhood deprivation (n=282 to 311)																
Total brain volume	-0.05	0.07	0.43	0.85	-0.03	0.09	0.72	0.72	0.14	0.07	0.04	0.30	**0.20**	**0.07**	**0.003**	**0.02**
Grey matter volume	-0.05	0.10	0.59	0.85	-0.08	0.14	0.56	0.72	0.04	0.10	0.72	0.80	**0.24**	**0.10**	**0.02**	**0.049**
Normal-appearing white matter volume	-0.03	0.10	0.74	0.85	0.15	0.13	0.25	0.72	0.07	0.10	0.53	0.80	0.07	0.11	0.49	0.79
White matter hyperintensity volume	0.10	0.16	0.51	0.85	-0.22	0.22	0.32	0.72	0.11	0.16	0.51	0.80	-0.02	0.17	0.92	0.92
Cortical surface area	0.01	0.09	0.94	0.94	-0.06	0.13	0.65	0.72	0.10	0.10	0.34	0.80	0.13	0.10	0.21	0.41
Mean cortical thickness	0.09	0.18	0.61	0.85	-0.10	0.25	0.69	0.72	0.05	0.19	0.80	0.80	0.06	0.20	0.76	0.87
General fractional anisotropy[a]	0.12	0.19	0.50	0.85	-0.11	0.26	0.67	0.72	0.23	0.19	0.23	0.80	**0.52**	**0.20**	**0.009**	**0.04**
General mean diffusivity[a]	-0.09	0.19	0.65	0.85	0.39	0.26	0.14	0.72	0.09	0.20	0.66	0.80	0.07	0.21	0.74	0.87
Young adulthood neighbourhood deprivation (n=348 to 383)																
Total brain volume	-0.07	0.06	0.24	0.83	-0.03	0.07	0.72	0.99	0.16	0.06	0.01	0.05	**0.20**	**0.06**	**0.002**	**0.007**
Grey matter volume	0.03	0.09	0.73	0.83	0.07	0.11	0.50	0.99	0.09	0.09	0.36	0.58	**0.34**	**0.09**	**<0.001**	**0.001**
Normal-appearing white matter volume	-0.15	0.09	0.10	0.81	0.00	0.12	0.98	0.99	0.11	0.10	0.29	0.58	-0.02	0.10	0.86	0.86
White matter hyperintensity volume	0.13	0.14	0.36	0.83	-0.44	0.17	0.01	0.09	0.06	0.15	0.66	0.75	0.17	0.15	0.26	0.35
Cortical surface area	0.00	0.08	0.97	0.97	-0.18	0.10	0.08	0.32	**0.24**	**0.09**	**0.005**	**0.04**	0.11	0.09	0.22	0.35
Mean cortical thickness	0.06	0.16	0.69	0.83	0.29	0.20	0.14	0.37	-0.12	0.16	0.48	0.64	0.39	0.17	0.02	0.06
General fractional anisotropy[a]	0.10	0.17	0.57	0.83	-0.06	0.21	0.77	0.99	0.32	0.18	0.07	0.17	0.35	0.18	0.06	0.11
General mean diffusivity[a]	0.10	0.17	0.58	0.83	0.00	0.22	0.99	0.99	0.02	0.18	0.92	0.92	0.09	0.19	0.64	0.73
Mid- to late adulthood neighbourhood deprivation (n=359 to 396)																
Total brain volume	0.01	0.06	0.90	0.97	-0.09	0.07	0.22	0.52	0.05	0.07	0.50	0.72	0.10	0.07	0.12	0.31
Grey matter volume	0.09	0.09	0.31	0.82	0.08	0.11	0.46	0.52	-0.01	0.10	0.89	0.89	0.25	0.10	0.01	0.08
Normal-appearing white matter volume	-0.10	0.09	0.26	0.82	-0.07	0.11	0.55	0.55	0.06	0.10	0.54	0.72	-0.17	0.10	0.09	0.31
White matter hyperintensity volume	0.09	0.14	0.50	0.97	-0.39	0.17	0.02	0.17	0.28	0.15	0.07	0.26	0.14	0.16	0.35	0.47
Cortical surface area	0.02	0.08	0.77	0.97	-0.08	0.10	0.44	0.52	0.23	0.09	0.01	0.06	0.00	0.09	0.98	0.98
Mean cortical thickness	0.01	0.16	0.97	0.97	-0.17	0.20	0.38	0.52	-0.18	0.17	0.30	0.60	0.10	0.18	0.57	0.65
General fractional anisotropy[a]	-0.06	0.17	0.71	0.97	-0.20	0.20	0.32	0.52	0.22	0.18	0.22	0.60	-0.24	0.19	0.19	0.39
General mean diffusivity[a]	0.20	0.17	0.25	0.82	-0.18	0.21	0.41	0.52	0.06	0.19	0.73	0.84	0.21	0.19	0.28	0.44
Accumulated neighbourhood deprivation (n=255 to 281)																
Total brain volume	-0.04	0.07	0.52	0.93	-0.10	0.09	0.30	0.57	0.15	0.08	0.04	0.17	**0.21**	**0.07**	**0.003**	**0.01**
Grey matter volume	-0.01	0.10	0.96	0.96	-0.11	0.15	0.43	0.57	0.12	0.12	0.30	0.50	**0.31**	**0.11**	**0.003**	**0.01**
Normal-appearing white matter volume	-0.05	0.10	0.62	0.93	0.12	0.15	0.40	0.57	0.12	0.12	0.31	0.50	-0.01	0.11	0.93	0.93
White matter hyperintensity volume	0.08	0.16	0.63	0.93	-0.44	0.22	0.05	0.36	0.15	0.18	0.41	0.54	0.17	0.17	0.34	0.55
Cortical surface area	0.04	0.10	0.67	0.93	-0.18	0.13	0.18	0.57	0.24	0.10	0.02	0.17	0.17	0.10	0.10	0.28
Mean cortical thickness	0.07	0.19	0.73	0.93	-0.18	0.28	0.51	0.58	-0.07	0.21	0.76	0.82	0.15	0.21	0.46	0.62
General fractional anisotropy[a]	0.23	0.20	0.25	0.93	-0.04	0.27	0.89	0.89	0.40	0.21	0.06	0.17	0.30	0.21	0.16	0.33
General mean diffusivity[a]	-0.05	0.21	0.82	0.93	0.27	0.29	0.36	0.57	0.05	0.23	0.82	0.82	0.10	0.23	0.65	0.77

Models with relevant interaction terms were fitted within the structural equation modelling framework applying full information maximum likelihood estimation. Total sample size was N=658 for total brain, grey matter and normal-appearing white matter volumes, N=672 for white matter hyperintensity volume, N=636 for cortical surface area and mean cortical thickness, and N=665 for general fractional anisotropy and mean diffusivity; this table indicates ranges of observations for each exposure-outcomes pairs on which reported effect sizes are based (n). Bold typeface denotes false discovery rate adjusted significance (p_{FDR}). SE = standard error.

Models were adjusted for sex, age, (intracranial volume for macrostructural measures,) father's occupational social class and APOE ε4 allele status. In addition, young adulthood models were adjusted for childhood IQ and years spent in education, and mid- to late adulthood/ accumulation models also for adult occupational social class.

[a] No adjustment for intracranial volume.

medRxiv preprint doi: https://doi.org/10.1101/2023.04.13.23288523; this version posted April 17, 2023. The copyright holder for this preprint (which was not certified by peer review) is the author/funder, who has granted medRxiv a license to display the preprint in perpetuity.
It is made available under a CC-BY 4.0 International license .

Supplementary Table 8: Association between life-course models of neighbourhood deprivation and global brain outcomes stratified by adult occupational social class

	**High: Professional-managerial**				**Low: Skilled, partly skilled, and unskilled**			
	β	SE	*p*	p_{FDR}	*β*	SE	*p*	p_{FDR}
Childhood neighbourhood deprivation	(*n*=168 to 173)				(*n*=126 to 136)			
Total brain volume	0.02	0.03	0.39	0.77	**-0.10**	**0.03**	**0.003**	**0.01**
Grey matter volume	0.01	0.04	0.76	0.88	**-0.12**	**0.05**	**0.02**	**0.05**
Normal-appearing white matter volume	0.04	0.04	0.35	0.77	-0.02	0.05	0.76	0.90
White matter hyperintensity volume	-0.02	0.07	0.77	0.88	-0.02	0.08	0.83	0.90
Cortical surface area	0.04	0.04	0.33	0.77	-0.04	0.05	0.36	0.63
Mean cortical thickness	-0.05	0.08	0.51	0.82	-0.08	0.09	0.39	0.63
General fractional anisotropy[a]	0.08	0.08	0.33	0.77	**-0.26**	**0.09**	**0.003**	**0.01**
General mean diffusivity[a]	0.00	0.08	0.99	0.99	0.01	0.10	0.90	0.90
Young adulthood neighbourhood deprivation	(*n*=220 to 226)				(*n*=144 to 154)			
Total brain volume	0.03	0.03	0.17	0.78	**-0.08**	**0.03**	**0.013**	**0.03**
Grey matter volume	0.04	0.04	0.29	0.78	**-0.15**	**0.05**	**0.002**	**0.005**
Normal-appearing white matter volume	0.05	0.04	0.24	0.78	0.05	0.05	0.27	0.31
White matter hyperintensity volume	0.03	0.06	0.62	0.85	-0.11	0.08	0.14	0.19
Cortical surface area	-0.01	0.04	0.74	0.85	-0.09	0.05	0.05	0.08
Mean cortical thickness	-0.01	0.07	0.87	0.87	**-0.25**	**0.08**	**0.002**	**0.005**
General fractional anisotropy[a]	-0.03	0.08	0.66	0.85	**-0.28**	**0.08**	**0.001**	**0.005**
General mean diffusivity[a]	-0.04	0.08	0.56	0.85	-0.04	0.10	0.65	0.65
Mid- to late adulthood neighbourhood deprivation	(*n*=233 to 240)				(*n*=144 to 154)			
Total brain volume	-0.03	0.03	0.19	0.31	**-0.09**	**0.03**	**0.006**	**0.022**
Grey matter volume	-0.04	0.04	0.26	0.32	**-0.18**	**0.05**	**<0.001**	**0.003**
Normal-appearing white matter volume	**-0.11**	**0.04**	**0.004**	**0.017**	-0.04	0.05	0.45	0.62
White matter hyperintensity volume	0.10	0.07	0.14	0.27	-0.01	0.08	0.94	0.94
Cortical surface area	-0.04	0.04	0.32	0.32	-0.04	0.05	0.46	0.62
Mean cortical thickness	-0.13	0.07	0.08	0.22	-0.19	0.09	0.03	0.09
General fractional anisotropy[a]	**-0.26**	**0.07**	**<0.001**	**0.003**	-0.12	0.09	0.19	0.38
General mean diffusivity[a]	0.08	0.08	0.32	0.32	-0.02	0.10	0.85	0.94
Accumulated neighbourhood deprivation	(*n*=147 to 151)				(*n*=120 to 129)			
Total brain volume	0.02	0.03	0.44	0.87	**-0.13**	**0.04**	**<0.001**	**0.001**
Grey matter volume	0.01	0.05	0.76	0.87	**-0.21**	**0.06**	**<0.001**	**0.001**
Normal-appearing white matter volume	0.02	0.05	0.69	0.87	0.01	0.05	0.79	0.85
White matter hyperintensity volume	0.05	0.08	0.56	0.87	-0.09	0.09	0.29	0.39
Cortical surface area	0.04	0.05	0.36	0.87	-0.09	0.05	0.10	0.15
Mean cortical thickness	-0.04	0.10	0.67	0.87	-0.19	0.10	0.06	0.13
General fractional anisotropy[a]	0.00	0.10	0.97	0.97	**-0.27**	**0.10**	**0.006**	**0.016**
General mean diffusivity[a]	-0.08	0.10	0.43	0.87	-0.02	0.11	0.85	0.85

Models were fitted within the structural equation modelling framework applying full information maximum likelihood estimation. Total sample size was *N*=376/*N*=272 for total brain, grey matter and normal-appearing white matter volume, *N*=383/*N*=278 for white matter hyperintensity volume, N=368/*N*=257 for cortical surface area and mean cortical thickness, and *N*=381/274 for general fractional anisotropy and mean diffusivity among high/low social classes; this table indicates ranges of observations for exposure-outcomes pairs (*n*). Bold typeface denotes false discovery rate adjusted significance (p_{FDR}). SE = standard error.

Models were adjusted for sex, age, (intracranial volume for macrostructural measures,) father's occupational social class and *APOE* ε4 allele status. In addition, young adulthood models were adjusted for childhood IQ and years spent in education, and mid- to late adulthood/ accumulation models also for adult occupational social class.

[a] No adjustment for intracranial volume

medRxiv preprint doi: https://doi.org/10.1101/2023.04.13.23288523; this version posted April 17, 2023. The copyright holder for this preprint (which was not certified by peer review) is the author/funder, who has granted medRxiv a license to display the preprint in perpetuity.
It is made available under a CC-BY 4.0 International license .

Supplementary Table 9: Association between life-course models of neighbourhood deprivation and global brain outcomes after considering exposure during previous epoch

	Model 2 – S1				% Change from Model 2[a]
	β	SE	*p*	*pFDR*	
Young adulthood neighbourhood deprivation (n=367 to 383)					
Total brain volume	0.01	0.02	0.69	0.78	-150.00%
Grey matter volume	-0.03	0.04	0.47	0.63	-40.00%
Normal-appearing white matter volume	0.07	0.04	0.08	0.21	40.00%
White matter hyperintensity volume	-0.02	0.06	0.78	0.78	-55.00%
Cortical surface area	-0.05	0.04	0.15	0.30	0.00%
Mean cortical thickness	-0.12	0.07	0.07	0.21	-20.00%
General fractional anisotropy[b]	-0.13	0.07	0.06	0.21	-7.14%
General mean diffusivity[b]	-0.09	0.07	0.22	0.35	50.00%
Mid- to late adulthood neighbourhood deprivation (n=379 to 396)					
Total brain volume	**-0.05**	**0.02**	**0.03**	**0.04**	**-16.67%**
Grey matter volume	**-0.09**	**0.03**	**0.01**	**0.03**	**-18.18%**
Normal-appearing white matter volume	**-0.09**	**0.03**	**0.01**	**0.03**	**28.57%**
White matter hyperintensity volume	0.07	0.05	0.22	0.29	40.00%
Cortical surface area	-0.01	0.03	0.67	0.76	-75.00%
Mean cortical thickness	**-0.15**	**0.06**	**0.02**	**0.03**	**0.00%**
General fractional anisotropy[b]	**-0.17**	**0.06**	**0.01**	**0.03**	**-10.53%**
General mean diffusivity[b]	0.01	0.07	0.86	0.86	-66.67%

Models were fitted within the structural equation modelling framework applying full information maximum likelihood estimation; exposure of interest was regressed on exposure during the previous period (i.e., young adulthood on childhood, mid- to late adulthood on young adulthood neighbourhood deprivation). Models for childhood neighbourhood deprivation and accumulated neighbourhood deprivation are not presented as this sensitivity analysis cannot be applied for them. Total sample size was *N*=658 for total brain, grey matter and normal-appearing white matter volumes, *N*=672 for white matter hyperintensity volume, *N*=636 for cortical surface area and mean cortical thickness, and *N*=665 for general fractional anisotropy and mean diffusivity; this table indicates ranges of observations for each exposure-outcomes pairs on which reported effect sizes are based (*n*). Bold typeface denotes false discovery rate adjusted significance (p_{FDR}). SE = standard error.

Models were adjusted for sex, age, (intracranial volume for macrostructural measures,) father's occupational social class, *APOE* ε4 allele status, childhood IQ, years spent in education and neighbourhood deprivation during previous exposure period, mid- to late adulthood models also adjusted for adult occupational social class.

[a] Main models did not include neighbourhood deprivation in previous epochs.
[b] No adjustment for intracranial volume.

medRxiv preprint doi: https://doi.org/10.1101/2023.04.13.23288523; this version posted April 17, 2023. The copyright holder for this preprint (which was not certified by peer review) is the author/funder, who has granted medRxiv a license to display the preprint in perpetuity.
It is made available under a CC-BY 4.0 International license .

Supplementary Table 10: Association between life-course models of neighbourhood deprivation and global brain outcomes after adjusting for stroke identified from MRI scans

	Model 2 – S2			
	β	SE	*p*	p_{FDR}
Childhood neighbourhood deprivation (n=296 to 311)				
Total brain volume	-0.04	0.02	0.10	0.39
Grey matter volume	-0.05	0.03	0.09	0.39
Normal-appearing white matter volume	0.02	0.03	0.58	0.77
White matter hyperintensity volume	-0.03	0.05	0.57	0.77
Cortical surface area	-0.01	0.03	0.84	0.96
Mean cortical thickness	-0.07	0.06	0.23	0.61
General fractional anisotropy[a]	-0.06	0.06	0.30	0.61
General mean diffusivity[a]	0.00	0.06	0.98	0.98
Young adulthood neighbourhood social deprivation (n=367 to 383)				
Total brain volume	-0.02	0.02	0.34	0.44
Grey matter volume	-0.05	0.03	0.11	0.22
Normal-appearing white matter volume	0.05	0.03	0.14	0.22
White matter hyperintensity volume	-0.03	0.05	0.48	0.48
Cortical surface area	-0.05	0.03	0.06	0.17
Mean cortical thickness	-0.10	0.05	0.06	0.17
General fractional anisotropy[a]	-0.14	0.06	0.01	0.11
General mean diffusivity[a]	-0.05	0.06	0.39	0.44
Mid- to late adulthood neighbourhood deprivation (n=379 to 396)				
Total brain volume	**-0.06**	**0.02**	**0.01**	**0.02**
Grey matter volume	**-0.10**	**0.03**	**0.002**	**0.009**
Normal-appearing white matter volume	-0.06	0.03	0.07	0.11
White matter hyperintensity volume	0.04	0.05	0.46	0.52
Cortical surface area	-0.04	0.03	0.16	0.21
Mean cortical thickness	**-0.15**	**0.06**	**0.01**	**0.02**
General fractional anisotropy[a]	**-0.18**	**0.06**	**0.002**	**0.009**
General mean diffusivity[a]	0.01	0.06	0.83	0.83
Accumulated neighbourhood deprivation (n=268 to 281)				
Total brain volume	-0.05	0.03	0.05	0.20
Grey matter volume	-0.09	0.04	0.02	0.19
Normal-appearing white matter volume	0.02	0.04	0.53	0.57
White matter hyperintensity volume	-0.03	0.06	0.57	0.57
Cortical surface area	-0.02	0.04	0.53	0.57
Mean cortical thickness	-0.11	0.07	0.15	0.29
General fractional anisotropy[a]	-0.13	0.07	0.08	0.22
General mean diffusivity[a]	-0.07	0.08	0.38	0.57

Models were fitted within the structural equation modelling framework applying full information maximum likelihood estimation. Total sample size was N=658 for total brain, grey matter and normal-appearing white matter volume, N=672 for white matter hyperintensity volume, N=636 for cortical surface area and mean cortical thickness, and N=665 for general fractional anisotropy and mean diffusivity; this table indicates ranges of observations for each exposure-outcomes pairs on which reported effect sizes are based (n). Bold typeface denotes false discovery rate adjusted significance (p_{FDR}). SE = standard error.

Models were adjusted for sex, age, MRI-based stroke, (intracranial volume for macrostructural measures,) father's occupational social class and *APOE* ε4 allele status. In addition, young adulthood models were adjusted for childhood IQ and years spent in education, and mid- to late adulthood / accumulation models also for adult occupational social class.

[a] No adjustment for intracranial volume

medRxiv preprint doi: https://doi.org/10.1101/2023.04.13.23288523; this version posted April 17, 2023. The copyright holder for this preprint (which was not certified by peer review) is the author/funder, who has granted medRxiv a license to display the preprint in perpetuity.
It is made available under a CC-BY 4.0 International license .

Supplementary Table 11: Association between mid-to-late adulthood neighbourhood deprivation and global brain outcomes after adjusting for late adulthood health status

	**Model 2 – S3**				**% Change from Model 2[a]**
	β	SE	*p*	p_{FDR}	
Mid- to late adulthood neighbourhood deprivation (n=379 to 396)					
Total brain volume	-0.05	0.02	0.02	0.05	-16.67%
Grey matter volume	**-0.09**	**0.03**	**0.004**	**0.02**	**-18.18%**
Normal-appearing white matter volume	-0.05	0.03	0.10	0.15	-28.57%
White matter hyperintensity volume	0.04	0.05	0.42	0.48	-20.00%
Cortical surface area	-0.04	0.03	0.20	0.27	0.00%
Mean cortical thickness	-0.10	0.06	0.07	0.15	-33.33%
General fractional anisotropy[b]	**-0.17**	**0.06**	**0.004**	**0.02**	**-10.53%**
General mean diffusivity[b]	0.02	0.06	0.71	0.71	-33.33%

Models were fitted within the structural equation modelling framework applying full information maximum likelihood estimation. Total sample size was *N*=658 for total brain, grey matter and normal-appearing white matter volumes, *N*=672 for white matter hyperintensity volume, *N*=636 for cortical surface area and mean cortical thickness, and *N*=665 for general fractional anisotropy and mean diffusivity; this table indicates ranges of observations for each exposure-outcomes pairs on which reported effect sizes are based (*n*). Bold typeface denotes false discovery rate adjusted significance (p_{FDR}). SE = standard error.

Models were adjusted for sex, age, (intracranial volume for volumetric measures,) father's occupational social class, *APOE* ε4 allele status, childhood IQ, years spent in education, adult occupational social class, smoking status, BMI, and history of hypertension, diabetes, stroke, and cardiovascular diseases.

[a] Main models did not include health-related variables.
[b] No adjustment for intracranial volume

medRxiv preprint doi: https://doi.org/10.1101/2023.04.13.23288523; this version posted April 17, 2023. The copyright holder for this preprint (which was not certified by peer review) is the author/funder, who has granted medRxiv a license to display the preprint in perpetuity.
It is made available under a CC-BY 4.0 International license .

Supplementary Table 12: Association between life-course models of neighbourhood deprivation and global brain outcomes among those living in Edinburgh throughout each decade of the exposure periods

	Model 2 – S4			
	β	SE	*p*	p_{FDR}
Childhood neighbourhood social deprivation (n=264 to 276)				
Total brain volume	-0.06	0.02	0.02	0.17
Grey matter volume	-0.06	0.04	0.10	0.39
Normal-appearing white matter volume	0.00	0.04	0.92	0.92
White matter hyperintensity volume	-0.03	0.06	0.64	0.85
Cortical surface area	-0.02	0.03	0.59	0.85
Mean cortical thickness	-0.07	0.07	0.32	0.64
General fractional anisotropy[a]	-0.08	0.07	0.25	0.64
General mean diffusivity[a]	-0.02	0.07	0.81	0.92
Young adulthood neighbourhood social deprivation (n=300 to 314)				
Total brain volume	-0.02	0.02	0.46	0.52
Grey matter volume	-0.03	0.04	0.46	0.52
Normal-appearing white matter volume	0.05	0.04	0.16	0.42
White matter hyperintensity volume	-0.05	0.05	0.39	0.52
Cortical surface area	-0.06	0.03	0.07	0.28
Mean cortical thickness	-0.02	0.07	0.72	0.72
General fractional anisotropy[a]	-0.12	0.07	0.06	0.28
General mean diffusivity[a]	-0.08	0.07	0.24	0.48
Mid- to late adulthood neighbourhood deprivation (n=333 to 350)				
Total brain volume	**-0.06**	**0.02**	**0.02**	**0.05**
Grey matter volume	**-0.10**	**0.03**	**0.004**	**0.03**
Normal-appearing white matter volume	-0.06	0.03	0.07	0.12
White matter hyperintensity volume	0.04	0.05	0.48	0.48
Cortical surface area	-0.03	0.03	0.45	0.48
Mean cortical thickness	**-0.16**	**0.06**	**0.01**	**0.04**
General fractional anisotropy[a]	**-0.15**	**0.07**	**0.02**	**0.05**
General mean diffusivity[a]	-0.05	0.07	0.46	0.48
Accumulated neighbourhood deprivation (n=216 to 226)				
Total brain volume	**-0.09**	**0.03**	**0.004**	**0.03**
Grey matter volume	-0.12	0.05	0.01	0.05
Normal-appearing white matter volume	-0.01	0.05	0.88	0.88
White matter hyperintensity volume	-0.03	0.07	0.66	0.75
Cortical surface area	-0.04	0.04	0.34	0.45
Mean cortical thickness	-0.12	0.09	0.17	0.34
General fractional anisotropy[a]	-0.16	0.09	0.08	0.20
General mean diffusivity[a]	-0.09	0.10	0.33	0.45

Models were fitted within the structural equation modelling framework applying full information maximum likelihood estimation. Total sample size was *N*=658 for total brain, grey matter and normal-appearing white matter volumes, *N*=672 for white matter hyperintensity volume, *N*=636 for cortical surface area and mean cortical thickness, and *N*=665 for general fractional anisotropy and mean diffusivity; this table indicates ranges of observations for each exposure-outcomes pairs on which reported effect sizes are based (*n*). Bold typeface denotes false discovery rate adjusted significance (p_{FDR}). SE = standard error.

Models were adjusted for sex, age, (intracranial volume for macrostructural measures,) father's occupational social class and *APOE* ε4 allele status. In addition, young adulthood models were adjusted for childhood IQ and years spent in education, and mid- to late adulthood/ accumulation models also for adult occupational social class.

[a] No adjustment for intracranial volume

medRxiv preprint doi: https://doi.org/10.1101/2023.04.13.23288523; this version posted April 17, 2023. The copyright holder for this preprint (which was not certified by peer review) is the author/funder, who has granted medRxiv a license to display the preprint in perpetuity.
It is made available under a CC-BY 4.0 International license .

Supplementary Table 13: Association between life-course models of neighbourhood deprivation and global brain outcomes among individuals without cognitive impairment

	Model 2 – S5			
	β	SE	*p*	*p_{FDR}*
Childhood neighbourhood social deprivation (n=246 to 255)				
Total brain volume	-0.05	0.02	0.01	0.10
Grey matter volume	-0.05	0.03	0.11	0.44
Normal-appearing white matter volume	0.00	0.03	1.00	1.00
White matter hyperintensity volume	-0.05	0.05	0.34	0.54
Cortical surface area	-0.01	0.03	0.70	0.80
Mean cortical thickness	-0.08	0.06	0.20	0.53
General fractional anisotropy[a]	-0.07	0.07	0.30	0.54
General mean diffusivity[a]	-0.03	0.07	0.61	0.80
Young adulthood neighbourhood social deprivation (n=280 to 292)				
Total brain volume	-0.03	0.02	0.22	0.28
Grey matter volume	-0.04	0.03	0.21	0.28
Normal-appearing white matter volume	0.04	0.03	0.24	0.28
White matter hyperintensity volume	-0.05	0.05	0.31	0.31
Cortical surface area	-0.06	0.03	0.04	0.15
Mean cortical thickness	-0.10	0.06	0.06	0.17
General fractional anisotropy[a]	**-0.17**	**0.06**	**0.005**	**0.04**
General mean diffusivity[a]	-0.09	0.06	0.17	0.28
Mid- to late adulthood neighbourhood deprivation (n=313 to 328)				
Total brain volume	**-0.07**	**0.02**	**0.003**	**0.01**
Grey matter volume	**-0.11**	**0.03**	**0.001**	**0.008**
Normal-appearing white matter volume	**-0.09**	**0.03**	**0.006**	**0.01**
White matter hyperintensity volume	0.05	0.05	0.39	0.44
Cortical surface area	-0.04	0.03	0.18	0.23
Mean cortical thickness	**-0.15**	**0.06**	**0.009**	**0.01**
General fractional anisotropy[a]	**-0.18**	**0.06**	**0.004**	**0.01**
General mean diffusivity[a]	0.03	0.07	0.69	0.69
Accumulated neighbourhood deprivation (n=201 to 209)				
Total brain volume	**-0.07**	**0.03**	**0.006**	**0.047**
Grey matter volume	-0.09	0.04	0.02	0.10
Normal-appearing white matter volume	-0.02	0.04	0.61	0.61
White matter hyperintensity volume	-0.04	0.06	0.50	0.58
Cortical surface area	-0.03	0.04	0.37	0.51
Mean cortical thickness	-0.12	0.08	0.13	0.26
General fractional anisotropy[a]	-0.15	0.08	0.06	0.15
General mean diffusivity[a]	-0.07	0.08	0.39	0.51

Models were fitted within the structural equation modelling framework applying full information maximum likelihood estimation. Cognitive impairment was defined as either having a diagnosis of dementia or scoring <24 points in the Mini Mental State Examination. Total sample size was *N*=611 for total brain, grey matter and normal-appearing white matter volume, *N*=625 for white matter hyperintensity volume, *N*=594 for cortical surface area and mean cortical thickness, and *N*=620 for general fractional anisotropy and mean diffusivity; ranges of observations for exposure-outcomes pairs are presented in the table (*n*). Bold typeface denotes false discovery rate adjusted significance (p_{FDR}). SE = standard error.

Models were adjusted for sex, age, (intracranial volume for macrostructural measures,) father's occupational social class and *APOE* ε4 allele status. In addition, young adulthood models were adjusted for childhood IQ and years spent in education, and mid- to late adulthood/ accumulation models also for adult occupational social class.

[a] No adjustment for intracranial volume

medRxiv preprint doi: https://doi.org/10.1101/2023.04.13.23288523; this version posted April 17, 2023. The copyright holder for this preprint (which was not certified by peer review) is the author/funder, who has granted medRxiv a license to display the preprint in perpetuity.
It is made available under a CC-BY 4.0 International license .

Supplementary Table 14: Complete case analysis testing the association between life-course models of neighbourhood deprivation and global brain outcomes

	Model 2 – S6			
	β	SE	*p*	p_{FDR}
Childhood neighbourhood deprivation				
Total brain volume (*N*=261)	-0.05	0.02	0.02	0.13
Grey matter volume (*N*=261)	-0.05	0.04	0.16	0.54
Normal-appearing white matter volume (*N*=261)	0.00	0.03	0.94	0.94
White matter hyperintensity volume (*N*=266)	-0.06	0.07	0.40	0.64
Cortical surface area (*N*=255)	-0.02	0.04	0.64	0.86
Mean cortical thickness (*N*=255)	-0.07	0.07	0.27	0.54
General fractional anisotropy[a] (*N*=263)	-0.08	0.07	0.25	0.54
General mean diffusivity[a] (*N*=263)	-0.02	0.06	0.81	0.92
Young adulthood neighbourhood deprivation				
Total brain volume (*N*=310)	-0.02	0.02	0.27	0.44
Grey matter volume (*N*=310)	-0.03	0.03	0.39	0.46
Normal-appearing white matter volume (*N*=310)	0.02	0.03	0.46	0.46
White matter hyperintensity volume (*N*=316)	-0.07	0.07	0.28	0.44
Cortical surface area (*N*=304)	-0.08	0.03	0.02	0.09
Mean cortical thickness (*N*=304)	-0.09	0.06	0.17	0.44
General fractional anisotropy[a] (*N*=311)	**-0.18**	**0.06**	**0.005**	**0.04**
General mean diffusivity[a] (*N*=311)	-0.05	0.06	0.41	0.46
Mid- to late adulthood neighbourhood deprivation				
Total brain volume (*N*=321)	-0.05	0.02	0.03	0.07
Grey matter volume (*N*=321)	-0.07	0.03	0.04	0.08
Normal-appearing white matter volume (*N*=321)	-0.07	0.03	0.01	0.06
White matter hyperintensity volume (*N*=326)	0.03	0.07	0.67	0.68
Cortical surface area (*N*=313)	-0.04	0.04	0.33	0.45
Mean cortical thickness (*N*=313)	-0.13	0.07	0.06	0.09
General fractional anisotropy[a] (*N*=321)	**-0.20**	**0.06**	**0.002**	**0.02**
General mean diffusivity[a] (*N*=321)	0.03	0.06	0.68	0.68
Accumulated neighbourhood deprivation				
Total brain volume (*N*=228)	-0.06	0.03	0.02	0.18
Grey matter volume (*N*=228)	-0.06	0.04	0.11	0.29
Normal-appearing white matter volume (*N*=228)	-0.01	0.04	0.70	0.70
White matter hyperintensity volume (*N*=232)	-0.06	0.09	0.52	0.60
Cortical surface area (*N*=222)	-0.04	0.04	0.36	0.49
Mean cortical thickness (*N*=222)	-0.07	0.08	0.37	0.49
General fractional anisotropy[a] (*N*=229)	-0.15	0.08	0.06	0.23
General mean diffusivity[a] (*N*=229)	-0.07	0.06	0.30	0.49

Models were fitted with multivariate linear regression applying complete case analysis; therefore, underlying sample sizes differ across the presented estimates. Latent factors of general fractional anisotropy and general mean diffusivity were predicted and extracted from the measurement model. Sample sizes for each model are indicated in the table (*N*). Bold typeface denotes false discovery rate adjusted significance (p_{FDR}). SE = standard error.

Models were adjusted for sex, age, (intracranial volume for macrostructural measures,) father's occupational social class and *APOE* ε4 allele status. In addition, young adulthood models were adjusted for childhood IQ and years spent in education, and mid- to late adulthood/ accumulation models also for adult occupational social class.

[a] No adjustment for intracranial volume

medRxiv preprint doi: https://doi.org/10.1101/2023.04.13.23288523; this version posted April 17, 2023. The copyright holder for this preprint (which was not certified by peer review) is the author/funder, who has granted medRxiv a license to display the preprint in perpetuity.
It is made available under a CC-BY 4.0 International license .

Supplementary Figure 8: Pearson's correlation coefficients between individual exposures to neighbourhood deprivation. Pairwise deletion was applied for missing values to preserve the maximum amount of information. Black rectangles indicate childhood (1936-1955), young adulthood (1956-1975), and mid- to late adulthood (1976-2005) periods. All presented correlation coefficients were significant ($p < 0.001$).

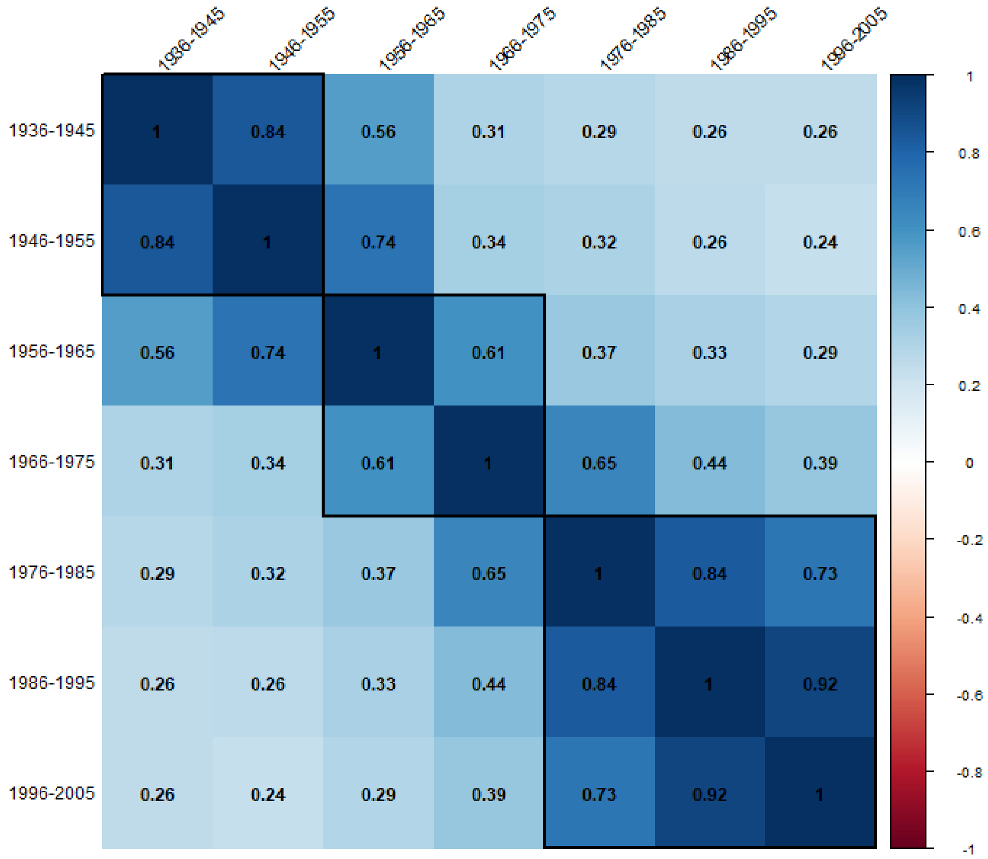

medRxiv preprint doi: https://doi.org/10.1101/2023.04.13.23288523; this version posted April 17, 2023. The copyright holder for this preprint (which was not certified by peer review) is the author/funder, who has granted medRxiv a license to display the preprint in perpetuity.
It is made available under a CC-BY 4.0 International license .

Supplementary Figure 9: Directed acyclic graph representing associations between neighbourhood deprivation, global brain outcomes and confounders considered in the main and sensitivity analyses. Confounders included in the main analyses are coloured grey: dark grey are considered as confounders for all life course models, medium grey for young adulthood and mid- to late adulthood exposures, light grey for mid- to late adulthood exposures only. Sensitivity analyses considering selective mobility (S1) and potential health-related confounders (S2, S3) are blue, red, and green, respectively. Links between confounders are not shown for simplicity. BMI = body mass index; IQ = intelligence quotient; ND = neighbourhood deprivation; OSC = occupational social class.

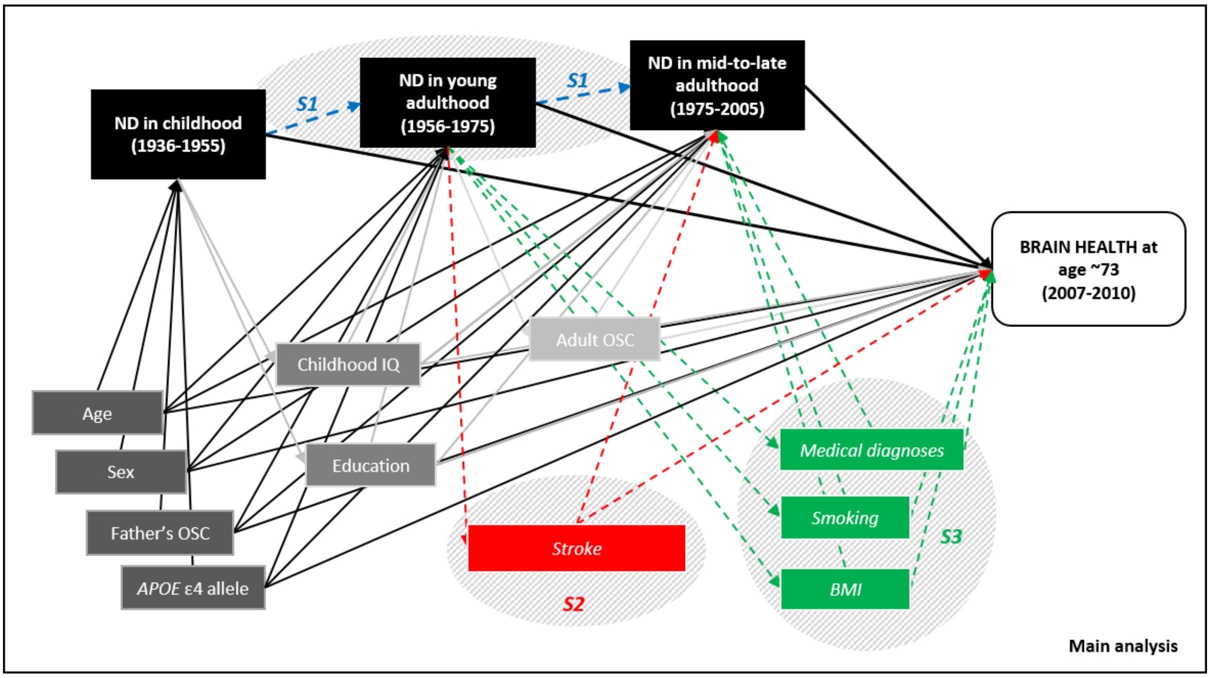

medRxiv preprint doi: https://doi.org/10.1101/2023.04.13.23288523; this version posted April 17, 2023. The copyright holder for this preprint (which was not certified by peer review) is the author/funder, who has granted medRxiv a license to display the preprint in perpetuity.
It is made available under a CC-BY 4.0 International license .

Supplementary Table 15: Standardized tract loadings, explained variance and goodness of fit indices for fractional anisotropy and mean diffusivity

	Fractional anisotropy	Mean diffusivity
Standardized tract loadings		
Genu of corpus callosum	0.57	0.57
Splenium of corpus callosum	0.41	0.28
Left arcuate fasciculus	0.65	0.70
Right arcuate fasciculus	0.65	0.74
Left anterior thalamic radiation	0.62	0.67
Right anterior thalamic radiation	0.60	0.58
Left rostral cingulum	0.52	0.60
Right rostral cingulum	0.53	0.72
Left inferior longitudinal fasciculus	0.46	0.37
Right inferior longitudinal fasciculus	0.44	0.36
Left uncinate fasciculus	0.62	0.63
Right uncinate fasciculus	0.65	0.68
% of variance explained		
	32.0%	35.2%
Goodness of fit indices		
Comparative Fit Index	0.97	0.97
Tucker Lewis Index	0.96	0.96
Root Mean Square Error of Approximation	0.04	0.05
Standardised Root Mean Square Residual	0.03	0.03
Chi-squared$_{(48)}$	106.34	131.43
p-value	<0.001	<0.001

Reference values are: Comparative Fit Index>0.95; Tucker-Lewis Index>0.95; Root Mean Square Error of Approximation<0.06; and Standardized Root Mean Square Residual<0.08 (1).

medRxiv preprint doi: https://doi.org/10.1101/2023.04.13.23288523; this version posted April 17, 2023. The copyright holder for this preprint (which was not certified by peer review) is the author/funder, who has granted medRxiv a license to display the preprint in perpetuity.
It is made available under a CC-BY 4.0 International license .

References

1. Hu Lt, Bentler PM. Cutoff criteria for fit indexes in covariance structure analysis: Conventional criteria versus new alternatives. Structural Equation Modeling: A Multidisciplinary Journal. 1999;6(1):1-55.

2. Lyall DM, Harris SE, Bastin ME, Muñoz Maniega S, Murray C, Lutz MW, et al. Alzheimer's disease susceptibility genes APOE and TOMM40, and brain white matter integrity in the Lothian Birth Cohort 1936. Neurobiol Aging. 2014;35(6):1513.e25-.e33.
